# X-ray nanotomography of dry colloidal packings

**DOI:** 10.1038/s41598-020-74315-2

**Published:** 2020-10-14

**Authors:** Yeseul Kim, Sangsul Lee, Jun Lim, Byung Mook Weon

**Affiliations:** 1 Soft Matter Physics Laboratory, SKKU Advanced Institute of Nanotechnology (SAINT), School of Advanced Materials Science and Engineering, Sungkyunkwan University, Suwon, Gyeonggi 16419 South Korea; 2 Industrial Technology Convergence Center, Pohang Light Source, Pohang, Gyeongbuk 37673 South Korea; 3 Research Center for Advanced Materials Technology, Sungkyunkwan University, Suwon, Gyeonggi 16419 South Korea; 4 Department of Biomedical Engineering, Johns Hopkins University, Baltimore, MD 21218 USA

**Keywords:** Structure of solids and liquids, Imaging techniques, Colloids

## Abstract

Random packings are crucial in understanding arrangement and geometry of particles. Random packings of dry small particles may be subject to adhesion or friction, as expected theoretically and numerically. We explore experimentally random packings of dry colloids with X-ray nanotomography that directly provides three-dimensional structural and geometric information of dry colloidal packings. We find that dry colloidal packings, as characterized by contact number and packing density, are quite consistent with adhesive loose packings that significantly deviate from random loose packings for hard spheres. This study may offer direct evidence for adhesive loose packings comprising dry small particles, as proven by X-ray nanotomography.

## Introduction

Random packings are natural and artificial agglomerates of spherical particles that generally occupy their volume fractions at $\phi \sim 0.56{-}0.64$^1–4^. In general, random packings of hard-sphere particles exist between liquid and crystal phases, showing unique features that are identical to glass and jamming phases^5,6^. For monodisperse hard spheres, the upper limit of random packings is called the random close packing (RCP) at $\phi _{RCP} \sim 0.64$ and the lower limit is the random loose packing (RLP) at $\phi _{RLP} \sim 0.56$^2–4^. The RCP structures commonly appear for frictionless spheres^7^, where the RLP structures for frictional spheres^8–11^. For colloidal suspensions and granular particles, random packings have long been well studied experimentally, numerically, and theoretically^1–16^. Random packings are common in colloidal particles (or colloids) that are small particles ranging from 10 nm to $10 \, \upmu \hbox {m}$ in diameter. Colloidal suspensions, where colloids are suspended in liquids, show typical characteristics of hard-sphere random packing aggregations^12,13^. For granular particles larger than several tens of $\upmu \hbox {m}$ in diameter, their random packings exhibit main features of hard-sphere random packings, regardless of their surrounding media^4,14,15^.

For dry small particles (or dry colloids), particularly without surrounding liquid media, their random packings are expected to deviate from hard-sphere random packings, as suggested numerically^17–24^. Because dry colloids are subject to adhesion and friction forces between colloids, their behaviors may deviate from hard spheres^25,26^. The adhesive loose packing (ALP) for dry small particles is expected to exist at the very low $\phi$ regime from the RLP phase ($\phi _{RLP} \sim 0.56$) up to the limit $\phi _{ALP} \sim (1/2)^{3}$, as previously suggested^21^. However, there is the lack of experimental evidence for the ALP structures of dry colloids.

In this study, to identify random packings of dry colloids, we adopted X-ray imaging techniques because they are powerful to characterize colloidal structures thanks to high-resolution and high-penetration capabilities of X-ray photons. Conventional imaging techniques such as electron microscopy (EM) or confocal laser microscopy (CLM) are not appropriate to visualize three-dimensional (3D) imaging of dry colloids. High vacuum for EM may deform random-packed colloidal packings. Big refractive index difference between air and colloids for CLM makes it impossible to image dry colloids^27^. To overcome this difficulty in imaging dry colloids, we used X-ray imaging that is irrelevant to deformation and big refractive index difference between air and colloids^28^. Additionally, X-ray imaging offers precise 3D structural information by applying tomographic techniques^9,28–30^. Using X-ray nanotomographic techniques, we are able to identify 3D structures of random packings from dry colloids and finally offer direct evidence for the ALP random structures of dry colloids. In particular, we quantify contact number and volume fraction of dry colloids with X-ray nanotomographic techniques.

## Methods

We explored the random packings of dry colloids with X-ray nonotomographic techniques. We used the full-field transmission X-ray microscopy (TXM) at the 7C X-ray Nano Imaging (XNI) beamline in the Pohang Light Source II (PLS-II). This beamline provided up to 40 nm spatial resolution with within a $150 \, \upmu \hbox {m}$ field of view^31^. By rotating the samples, TXM enabled us to achieve X-ray nanotomographic data as well. The hard X-ray beam with 6.7 keV photon energy penetrated random packings of silica particles. We prepared silica particles without treatments as purchased (L16985, L16986, L16987, Alfa Aesar, USA) with the particle diameters of 0.5, 1.0, and $1.5 \, \upmu \hbox {m}$ as model colloids. They were packed inside a thin borosilicate glass tube that was pulled by a commercial puller (Narishige PC-10, Japan) to be a thinner container with the diameter to $\sim 10{-}16 \, \upmu \hbox {m}$, appropriate to the fields of view of X-ray imaging. The thin glass tube was modified from the original tube (GC-1, Narishige Co., Japan) with the outer diameter and the thickness of 1.0 and 0.2 mm, respectively. Random packing of silica particles were fabricated by two different methods: dry colloids were manually packed by hands or mechanically packed with a centrifuge from a suspension in DI water and dried in still air. In turn, the randomly packed sample was mounted on a stage for X-ray imaging. After penetrating the sample, X-ray was converted into visible light by scintillator and captured by 20$\times$ objectives and CCD (Apogee Imaging Systems, U16MF, USA)^31^. We obtained X-ray nanotomographic image data set by acquiring 361 equiangular projections between 0$^{\circ }$ and $180^{\circ }$. The obtained phase contrast images were processed by subtracting the background. To improve the image quality, we tried to get sinograms based on the previously established method^32^. Then, the 3D reconstruction was performed with the OCTOPUS software (version 8.9.3) available at www.woorimtech.com/page/octopus411. The reconstruction into volume segmentation^33^ and the rendering process were performed with the Avizo software (version 9.0.1) available at https://www.fei.com/software/avizo3d/. The rendered images were created by the Avizo software and the Ovito software (version 2.9.0) available at https://www.ovito.org/about/version-history/.

## Results and discussion

**Figure 1:**
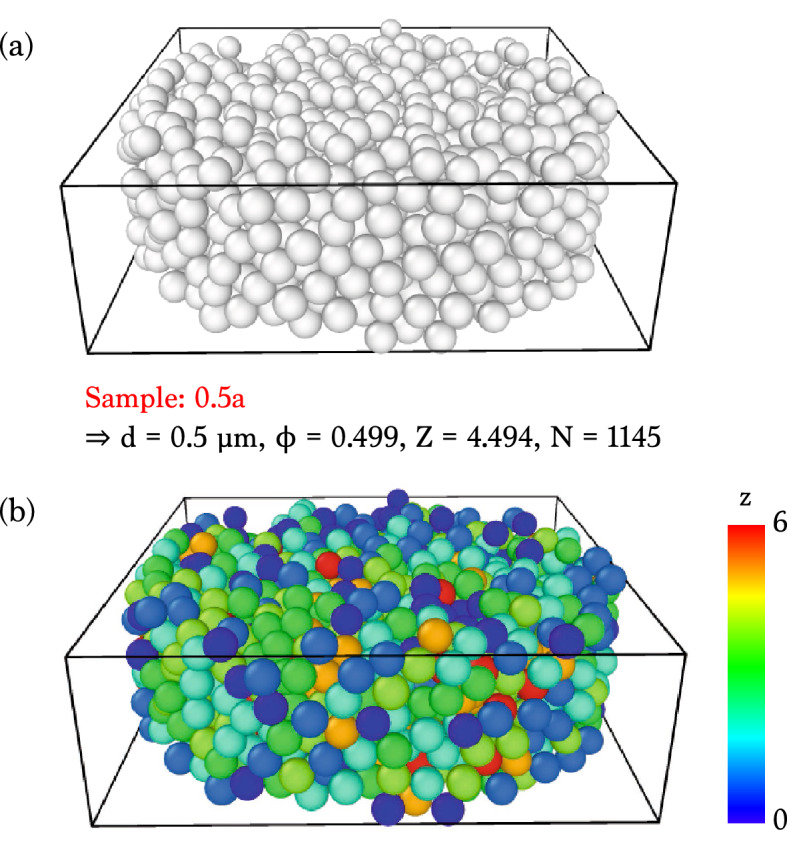
X-ray nanotomography of randomly-packed dry colloids. (**a**) 3D rendering image of the sample 0.5a, where the $0.5\hbox {-}\upmu \hbox {m}$-diameter silica particles are randomly packed. (**b**) Visualization of the local contact number (*z*) distribution by color based on X-ray nanotomographic analysis. Here, the 81 particles ($\sim 7.1\%$) of the entire 1145 particles are the rattlers with the zero local contact number ($z = 0$). The 3D reconstruction and the rendering process were performed with the OCTOPUS software (version 8.9.3) available at www.woorimtech.com/page/octopus411 and the Avizo software (version 9.0.1) available at https://www.fei.com/software/avizo3d/ and the rendered images were created by the Avizo software and the Ovito software (version 2.9.0) available at https://www.ovito.org/about/version-history/.

X-ray nanotomographic observations of dry colloidal packings directly enable us to explore the 3D structural information, as representatively illustrated in Fig. 1. The random packing consisting of the $0.5\hbox {-}\upmu \hbox {m}$-diameter colloids were prepared to have the volume fraction $\phi = 0.499$, the global contact number $Z = 4.494$, and the total number of particles $N = 1145$ (the sample 0.5a in Table 1), as confirmed by X-ray nanotomography. Here, the individual dry colloids are clearly distinguishable by X-ray nanotomography, as seen in Fig. 1a. Since the original X-ray 3D-rendered image has grey values (intensity from 0 to 65,535), it has to be converted to binary values (0 and 1) for analysis. Following the algorithm successfully used for identifying spheres^33^, we binarized the entire colloids except the two layers of colloidal particles from the boundaries, measured the particle diameters, and identified the positions from the centers of the particles. By adding the geometry information for the individual colloids, the dry colloidal packing was reconstructed, as demonstrated in Fig. 1a. Through a series of the image processing, the resulting image supports the usefulness of high-resolution X-ray nanotomography for the clear visualization of random packings of dry colloids without any structural destructions or interventions.

**Table 1:** Samples and X-ray nanotomographic data.

Name	Method	*d* ($\pm \sigma$) ($\upmu \hbox {m}$)	$\phi$	*Z*	*N*
0.5a	Centrifuge	0.5591 ($\pm 0.0129$)	0.499	4.494	1145
0.5b	Centrifuge	0.5452 ($\pm 0.0135$)	0.559	5.511	2767
0.5c	Manual	0.5428 ($\pm 0.0294$)	0.517	4.601	6342
1.0a	Manual	0.9848 ($\pm 0.0217$)	0.413	3.773	1873
1.0b	Manual	1.0043 ($\pm 0.0236$)	0.471	3.473	770
1.0c	Manual	0.9953 ($\pm 0.0267$)	0.545	5.052	476
1.5a	Centrifuge	1.4769 ($\pm 0.0342$)	0.497	4.646	692
1.5b	Centrifuge	1.4649 ($\pm 0.0295$)	0.502	4.940	1173
1.5c	Centrifuge	1.4455 ($\pm 0.0416$)	0.540	5.526	1750
1.5d	Manual	1.4405 ($\pm 0.0271$)	0.506	3.933	143
1.5e	Manual	1.4823 ($\pm 0.0427$)	0.484	3.397	444
1.5f	Manual	1.4835 ($\pm 0.0379$)	0.541	4.430	393

The randomly packed samples were prepared manually or mechanically with a centrifuge and analyzed with X-ray nanotomography to characterize the average particle diameter *d* ($\pm \sigma$, the standard deviation), the volume fraction $\phi$, the global contact number *Z*, and the total number of particles *N*.

Additionally, from the exact positions of the individual colloids, the local contact numbers of the individual colloids can be identified. The local contact numbers are the number of contacts with the neighboring colloids that exist within $\pm 5 \%$ of the colloid-colloid distance. The local contact numbers *z* are visualized at the individual colloids by color (from red $z = 6$ to blue $z = 0$) in Fig. 1b. X-ray nanotomography and image analysis techniques are powerful to visualize the distribution of the local contact numbers. From this image, we are able to see how the local contacts are randomly distributed in the dry colloidal packing. Here, the dry colloidal packing is stable at least during sample preparation and X-ray image acquisition time (${\sim } 2$ h). As a result, the dry colloids are stably packed and rarely segregated in space.

**Figure 2:**
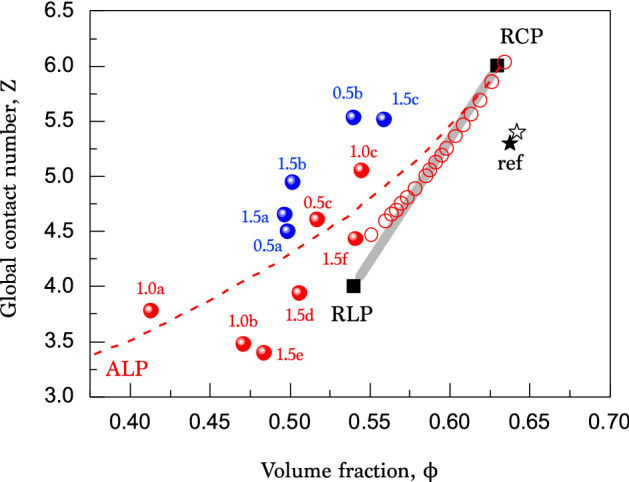
Dry colloidal packings with respect to global contact number and volume fraction. The data taken from Table 1 with X-ray nanotomography significantly deviate from the expectation for hard spheres between the random closed packing (RCP) and the random loose packing (RLP), consistent with the simulation results^35^ (red open circles). The samples were packed manually (red solid circles) and mechanically with a centrifuge (blue solid circles). The validity of the measurement is confirmed with the reference data^33^ (closed and open stars). The data are close to trend line of the adhesive loose packings (dashed line) taken from Ref.^21^.

X-ray naotomographic analyses for the 12 samples directly provide the geometric information such as the average particle diameter *d*, the volume fraction $\phi$, the global contact number *Z*, and the total number of particles *N*, based on the 3D structural and positional information, as summarized in Table 1. The global contact number is the contact number obtained by fitting the number of interactions versus the virtual diameters which was measured from the pair correlation function, by following the established method^33^. The *Z* value is estimated from the whole ensemble of colloids at once using their center of mass coordinates. Using the information from the ensemble of all colloids, it is possible to determine the best estimate for the colloid diameter itself from the pair correlation function and then to fit the contact number scaling (CNS) model to the data, allowing to identify the *Z* value^33^. The spatial resolution of X-ray nanotomography was ${\sim } 40 \, \hbox {nm}$ and much less than the particle diameter (0.5–1.5   $\upmu \hbox {m}$). In principle, the global contact number is crucial in understanding the stability of a random packing: for instance, a stable packing has $Z \ge 4.0$ for particles with friction, as commonly in granular media^34^. In our experiments, the actual *Z* values range from 3.397 to 5.526 (Table 1). The experimental determination of the *Z* values is feasible with X-ray nanotomography for dry colloids.

The volume fraction $\phi$ is an essential quantity achieved with X-ray nanotomography^36^. The total volume of the colloids *v* is measured by counting the total voxels with X-ray nanotomography. By dividing *v* by the cylinder volume *V* taken from X-ray nanotomography, we measure the volume fraction of the colloids as $\phi = v/V$. In our experiments, the $\phi$ values range from 0.413 to 0.559 (Table 1). Quantifying the $\phi$ values is crucial in identifying the nature of the dry colloidal packings.

The global contact number *Z* and the volume fraction $\phi$, taken with X-ray nanotomography, reveal the nature of the dry colloidal random packings. As summarized in Fig. 2, the dry colloidal packings significantly deviate from the hard-sphere random packings (from RCP $\phi _{RCP} \sim 0.64$ to RLP $\phi _{RLP} \sim 0.55$). Regardless the compaction process (manually or mechanically), the random packings of the dry colloids have the smaller volume fractions as $\phi \le 0.55$ ($0.413 \sim 0.559$). This result shows that the real volume fraction and the corresponding global contact number of the dry colloidal packings can exist in the lower quantities than expected. To show the validity of our analysis, we analyze the same reference data^33^, as marked by the stars in Fig. 2. The reference dataset is taken for a granular packing analyzed with X-ray microtomography and a Python code, showing $Z = 5.400$ and $\phi = 0.642$ (the open star). The same image is analyzed with our Labview code, resulting in $Z = 5.299$ and $\phi = 0.638$ (the closed star). The good agreement between two analyses verifies the validity of our analysis (but does not confirm that the phenomenological findings are artifact free).

**Figure 3:**
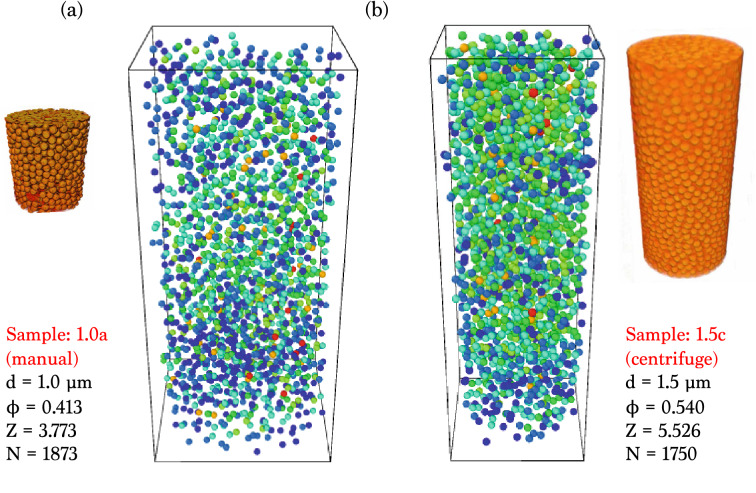
X-ray nanotomography of randomly packed dry colloidal packings. Two samples (1.0a and 1.5c in Table 1) have the similar total number of particles (*N*) but the different packing properties (*Z* and $\phi$): (**a**) an adhesive loose packing ($Z = 3.773$ and $\phi = 0.413$) and (**b**) a random loose packing ($Z = 5.526$ and $\phi = 0.540$). The 3D reconstruction and the rendering process were performed with the OCTOPUS software (version 8.9.3) available at www.woorimtech.com/page/octopus411 and the Avizo software (version 9.0.1) available at https://www.fei.com/software/avizo3d/ and the rendered images were created by the Avizo software and the Ovito software (version 2.9.0) available at https://www.ovito.org/about/version-history/.

The above experimental results suggest that the dry colloidal packings may significantly deviate from the RLP-RCP structures. Presumably, this deviation is attributed to the contribution of adhesion or friction to the packing stability. In fact, silica particles are known as having frictional performance^37^. The adhesion-controlled random packing of dry small particles, called the adhesive loose packing (ALP), was numerically studied in the recent work by Liu et al.^21–23^. The data of the dry colloidal packings seem to follow the ALP trend line (by fitted with the exponential model in Ref.^21^) as marked by the dashed line in Fig. 2.

The dry colloidal packings from two samples (1.0a and 1.5c in Table 1) are compared in Fig. 3. The packing characteristics depend on the diversity of sample preparation. X-ray nanotomography provides the 3D distributions of the local contact numbers for the random packings without any structural destructions and interventions. The dry colloids are stably clustered as the relatively loose random packings. The first structure in Fig. 3a shows the volume fraction $\phi = 0.413$, the global contact number $Z = 3.773$, and the particle number $N = 1873$, suggesting the ALP structure. The second structure in Fig. 3b shows the volume fraction $\phi = 0.540$, the global contact number $Z = 5.526$, and the particle number $N = 1750$, suggesting the RLP structure. These two packings are stable and demonstrating the random distributions of the local contacts.

**Figure 4:**
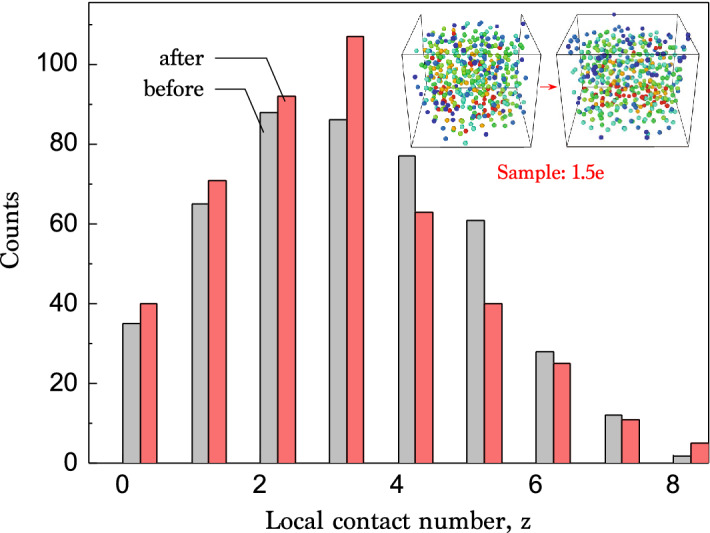
Local contact number distribution of dry colloidal packing before and after water addition and evaporation. Little difference between before and after water evaporation indicates little contribution of water to dry colloidal packings, suggesting little charging effects, agreeing with the global contact number change from 3.397 to 3.280. The 3D reconstruction and the rendering process were performed with the OCTOPUS software (version 8.9.3) available at www.woorimtech.com/page/octopus411 and the Avizo software (version 9.0.1) available at https://www.fei.com/software/avizo3d/ and the rendered images were created by the Avizo software and the Ovito software (version 2.9.0) available at https://www.ovito.org/about/version-history/.

**Figure 5:**
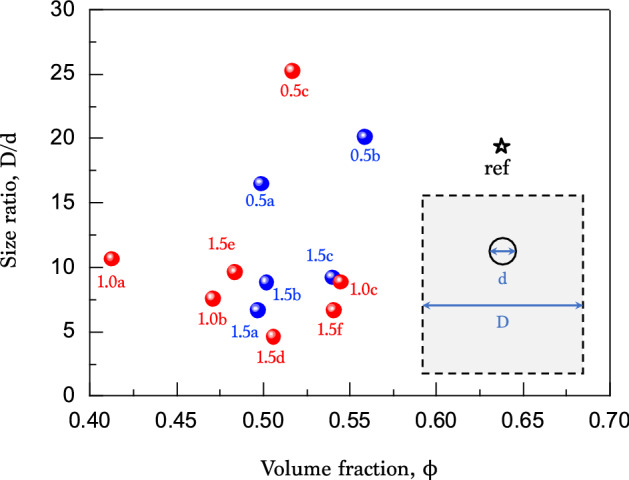
Little correlation between size ratio and volume fraction. The size ratio *D*/*d* from the cylinder diameter *D* and the colloid diameter *d* is almost independent of the volume fraction $\phi$, suggesting little wall effects.

The packing stability can be induced by electrostatic forces (charging effects)^38^, wall effects^16^, and van der Waals forces^26^, etc. To examine the first possibility of charging effects, we try to add water into the packing, which may affect the stability of the dry colloidal packing by compensating the possible charges. A small amount of water is added into the packed dry colloids and compared with X-ray nanotomography before and after water addition and evaporation, as shown in Fig. 4. Here is little change in their structures before and after water addition and evaporation. This result suggests that water does not affect the packing stability, implying that charging effects are negligible in the packing stability. The second possibility of wall effects is examined in Fig. 5. The size ratio *D*/*d* from the cylinder diameter *D* and the colloid diameter *d* is described with respect to the volume fraction $\phi$. If there exist significant wall effects, any correlations between *D*/*d* and $\phi$ can be observed for $D/d < 5.6$^16^. However, out data are quite diverse and larger than the criterion for wall effects as $D/d > 5.6$. This result suggests that wall effects are negligible in the packing stability. Finally, we may consider van der Waals forces for the adhesive loose packings^20,26^. These forces may be commonly dominate in the interactions between colloids smaller than $10 \, \upmu \hbox {m}$ in diameter^25,26^. For dry colloids (0.5–$1.5 \, \upmu \hbox {m}$ in diameter) examined in our experiments, the van der Waals forces are plausibly responsible for the packing stability. Further studies are required to corroborate the interparticle forces and the particle surface properties (roughness, chemistry, and charge), which may be crucial to dry colloidal packings.

## Conclusion

The random packings of dry small particles broadly appear in physics, chemistry, biology, agriculture, and engineering. The observations of dry colloidal packings with X-ray nanotomography suggest that the dry colloidal packings show the characteristics of the adhesive loose packings, as previously proposed. The actual packings of the equal-sized dry colloids with the diameters from 0.5 to $1.5 \, \upmu \hbox {m}$ are clearly visualized and analyzed with X-ray nanotomography. The global contact number and the volume fraction of the dry colloidal packings reveal the significant differences from the hard-sphere random loose or close packings. This discrepancy is attributed to the contribution of adhesion or friction forces inside dry colloids. This result may offer direct evidence for the adhesive loose packings comprising dry colloids. This study with X-ray nanotomography guides a feasible way to characterize complicated structures of dry small particles that are subject to adhesion or friction forces, which would be useful in a variety of natural and industrial applications.

## Data Availability

All data generated or analysed during this study are included in this published article.
